# The impact of patient registration on utilisation and quality of care: a propensity score matching and staggered difference-in-differences analysis of a cohort of 16,775 people with type 2 diabetes

**DOI:** 10.1186/s12875-024-02505-2

**Published:** 2024-07-12

**Authors:** Valerie Moran, Michela Bia, Patrick Thill, Marc Suhrcke, Ellen Nolte, Eric Burlot, Guy Fagherazzi

**Affiliations:** 1 Socio-Economic and Environmental Health and Health Services Research Group, Department of Precision Health, Luxembourg Institute of Health, Strassen, Luxembourg; 2 Socio-Economic and Environmental Health and Health Services Research Group, Living Conditions Department, Luxembourg Institute of Socio-Economic Research, Belval, Luxembourg; 3 Labour Market Department, Luxembourg Institute of Socio-Economic Research, Belval, Luxembourg; 4 Department of Health Services Research and Policy, London School of Hygiene and Tropical Medicine, London, UK; 5 Nomenclature, Conventions, Analysis and Forecasting Department, National Health Fund, Luxembourg, Luxembourg; 6 Deep Digital Phenotyping Research Unit, Department of Precision Health, Luxembourg Institute of Health, Strassen, Luxembourg

**Keywords:** Primary care reform, Patient registration, Type 2 diabetes, Health insurance claims data, Propensity score matching, Staggered difference-in-differences

## Abstract

**Background:**

In 2012, Luxembourg introduced a Referring Doctor (RD) policy, whereby patients voluntarily register with a primary care practitioner, who coordinates patients’ health care and ensures optimal follow-up. We contribute to the limited evidence base on patient registration by evaluating the effects of the RD policy.

**Methods:**

We used data on 16,775 people with type 2 diabetes on oral medication (PWT2D), enrolled with the Luxembourg National Fund from 2010 to 2018. We examined the utilisation of primary and specialist outpatient care, quality of care process indicators, and reimbursed prescribed medicines over the short- (until 2015) and medium-term (until 2018). We used propensity score matching to identify comparable groups of patients with and without an RD. We applied difference-in-differences methods that accounted for patients’ registration with an RD in different years.

**Results:**

There was low enrolment of PWT2D in the RD programme. The differences-in-differences parallel trends assumption was not met for: general practitioner (GP) consultations, GP home visits (medium-term), HbA1c test (short-term), complete cholesterol test (short-term), kidney function (urine) test (short-term), and the number of repeat prescribed cardiovascular system medicines (short-term). There was a statistically significant increase in the number of: HbA1c tests (medium-term: 0.09 (95% CI: 0.01 to 0.18)); kidney function (blood) tests in the short- (0.10 (95% CI: 0.01 to 0.19)) and medium-term (0.11 (95% CI: 0.03 to 0.20)); kidney function (urine) tests (medium-term: 0.06 (95% CI: 0.02 to 0.10)); repeat prescribed medicines in the short- (0.19 (95% CI: 0.03 to 0.36)) and medium-term (0.18 (95% CI: 0.02 to 0.34)); and repeat prescribed cardiovascular system medicines (medium-term: 0.08 (95% CI: 0.01 to 0.15)). Sensitivity analyses also revealed increases in kidney function (urine) tests (short-term: 0.07 (95% CI: 0.03 to 0.11)) and dental consultations (short-term: 0.06, 95% CI: 0.00 to 0.11), and decreases in specialist consultations (short-term: -0.28, 95% CI: -0.51 to -0.04; medium-term: -0.26, 95% CI: -0.49 to -0.03).

**Conclusions:**

The RD programme had a limited effect on care quality indicators and reimbursed prescribed medicines for PWT2D. Future research should extend the analysis beyond this cohort and explore data linkage to include clinical outcomes and socio-economic characteristics.

**Supplementary Information:**

The online version contains supplementary material available at 10.1186/s12875-024-02505-2.

## Introduction

Ageing populations and a rising burden of chronic diseases are creating a pressing need for countries to rethink service delivery approaches, away from an emphasis on specialist care and service fragmentation towards a more coordinated approach to meet the requirements of people with multiple or complex care needs associated with chronic disease. Failure to coordinate services along the care continuum may result in suboptimal outcomes, such as potentially preventable hospitalisations, medication errors, and other adverse events. Many countries have sought to strengthen the role of primary care as a hub for care coordination [1]. Patient registration with a primary care provider, with or without the requirement of referral from primary to secondary care (gatekeeping), can be seen as an important means to achieve improved coordination [2], and is widely considered as a key feature of strong primary care systems [3, 4].

Luxembourg has a single National Health Fund (Caisse nationale de santé). Enrollees have a free choice of doctor and there are no mandatory registration or gatekeeping systems. Doctors are paid on a fee-for-service basis. The National Health Fund reimburses 88% of the cost of a consultation with a general practitioner (GP) or specialist doctor [5]. Outpatient prescribed medicines on the ‘positive list’ are divided into three categories, which are reimbursed at different rates: 40% for medicines for the treatment of benign pathologies, 100% for medicines with a specific therapeutic indication (including medicines for the treatment of chronic conditions), and 80% for all other medicines [6]. Prescribed laboratory tests are fully reimbursed by the National Health Fund [7]. Luxembourg is one of the few European countries with a system of retrospective reimbursement, whereby patients pay the full cost upfront when they receive care and then claim reimbursement from the National Health Fund for the covered part of the cost. Outpatient prescribed medicines, prescribed laboratory tests and inpatient hospital care are exempt from retrospective reimbursement. In 2024, a system of ‘Direct Immediate Payment’ was introduced whereby doctors can receive immediate payment for the covered services directly from the National Health Fund, subject to consent from both the doctor and patient [8].

In 2012, Luxembourg introduced a voluntary form of patient registration with a primary care provider: the *médecin référent* or Referring Doctor (RD) programme. Only doctors who have undertaken specialty training in general practice or paediatrics and who provide primary care outside of a hospital setting are eligible to become an RD [9]. The role of the RD is to coordinate patients’ care, manage and monitor their conditions and guide patients towards other health professionals (such as medical specialists) as necessary. There was an expectation by the national government that this new role would contribute to optimising the use of medicines and preventing unnecessary consultations and investigations [9]. The RD does not act as a gatekeeper and patients who have registered with an RD can still consult a specialist directly. Participation in the programme is voluntary for both providers and eligible patients. Doctors receive an additional payment for participating in the programme and can charge the RD tariff six months following a patient declaration of participation in the RD programme. Only one payment can be claimed by the doctor every six months. Doctors do not receive any capitation payment for patients enrolled in the RD programme. Doctors and patients participating in the programme also need to activate their eHealth accounts in order to gain access to patients’ electronic health record [9, 10]. Initially, all enrollees of the National Health Fund were eligible to join the scheme, but from 2016, eligibility was restricted to people with specific chronic conditions as defined by a list agreed between the National Health Fund and the Luxembourgish Association of Doctors and Dentists (Association des Médecins et Médecins-Dentistes) [11].

Although other countries, such as France and Germany, have introduced voluntary patient registration with a primary care provider to strengthen care coordination, the RD policy implemented in Luxembourg differs in that it does not require or incentivise registered patients to obtain an RD referral to access a specialist. There is so far little robust evidence of the effects of patient registration within countries [2]. This work aims to address this important research gap. We evaluated the effect of the RD programme on health care utilisation, quality of care process indicators and reimbursed prescribed medicines, using health insurance data to compare the outcomes of people with type 2 diabetes who did and did not register with an RD.

## Methods

### Data

We used individual-level pseudonymised data from the Luxembourg National Health Fund to evaluate the RD programme during 2010–2018. This database contained information on all health services reimbursed by the National Health Fund for residents of Luxembourg who received health care services delivered in Luxembourg only. We focused on people with type 2 diabetes, who comprise the majority (over 90%) of diabetes patients worldwide [12]. Moreover, type 2 diabetes has a well-established evidence base for effective treatment that can be delivered in primary care [13]. There were no other national policy changes or reforms affecting the treatment of type 2 diabetes during the study period.

The database did not contain complete data on diagnosis. Instead, we used data on reimbursed prescribed medicines to infer patients’ type 2 diabetes diagnosis following Renard et al. (2011) [14] (see S1 Text). This identified 16,775 people who had reimbursed prescribed oral medicines for diabetes during 2010 and 2012 (subsequently referred to as people with type 2 diabetes, or PWT2D). We conducted a sensitivity analysis to expand our sample to include an additional 6,769 patients who had reimbursed prescribed oral medication and insulin. PWT2D can receive insulin treatment, if they fail to respond to oral or injectable antidiabetic agents [15].

## Variables

### Exposure variable

Exposure was defined as the year a patient first entered the RD programme as identified from the programme billing codes.

### Outcome measures

Our outcome measures included use of GP and specialist care, quality of care process indicators, and prescribed medicines, which we measured annually at an individual-level (S1 Table).

### Use of general practitioner (GP) and specialist care

We constructed two variables to measure utilisation of GP care to differentiate between (i) consultations with the GP in the GP practice and (ii) visits by the GP to a patient in the patients’ place of residence or in a hospital. As for specialist consultations, we distinguished those with a cardiologist from other specialist consultations. This is because diabetes guidelines in Luxembourg recommend an annual consultation with a cardiologist [16]. We were unable to consider endocrinologists separately to other specialists, as there was no separate billing code for endocrinologists until 2017. We hypothesised that the RD programme would lead to an increase in GP activity (consultations and visits). It is more difficult to predict the direction of the effect on specialist consultations: utilisation might decrease where the RD acts as ‘informal’ gatekeeper and filters demand for specialist care or substitutes some tasks; equally, it might increase where the RD identifies additional need for specialist care.

### Quality of care process indicators

We considered indicators included in Luxembourgish and international clinical guidelines for type 2 diabetes [13, 16, 17], specifically: (i) HbA1c measurement two to four times per year; annual (ii) cholesterol test (total cholesterol, HDL, LDL and triglycerides), (iii) creatinine with estimated glomerular filtration rate (hereafter kidney function (blood) test), (iv) albumin-to-creatinine ratio (hereafter kidney function (urine) test), (v) fundoscopic exam by an ophthalmologist and (vi) dental consultation. We excluded 2010 data for laboratory tests, as reimbursement for tests was restricted until 2010, artificially increasing the number of tests from 2011. We expect that the RD programme would improve quality of care process indicators.

### Reimbursed prescribed medicines

We constructed six variables to measure ‘continuous polypharmacy’ as defined by at least three reimbursed prescriptions of the same medicine in a year [18–20]. One variable measured the total number of medicines, and the remaining five variables measured the number of medicines for different comorbidities pertinent to type 2 diabetes, based on Anatomical Therapeutic Code (ATC) B (Blood and blood forming organs), C (Cardiovascular system), D (Dermatologists), N (Nervous system) and S (Sensory organs) [21]. While the RD policy may improve medication management and reduce polypharmacy, the programme may help to identify additional chronic conditions and lead to an increase in the prescribing of medicines for different comorbidities.

### Additional outcomes

We also considered additional outcomes that were not directly related to the RD programme objectives, but could be affected by the programme. These included urgent or out-of-hours care, hospital care, costs, and continuity of care, which we conceptualised as the number of different GPs reimbursed for the same patient (S1 Table).

### Control variables

We included year of birth (continuous), sex (male/female), civil status (married/civil partnership, single, separated/divorced, and widow(er)) and canton of residence as control variables, in order to control for observable differences that could explain registration with an RD.

### Statistical analysis

We used propensity score matching and difference-in-differences analysis to evaluate the effects of the RD policy, by comparing changes over time between PWT2D who did and did not register with an RD. Propensity score matching controls for observable characteristics that predict the probability of an individual registering with an RD. Following Stuart et al. (2014) [22], we used propensity score matching based on the nearest neighbour approach with replacement, whereby an individual registered with an RD is matched to the nearest (with the smallest distance) individual who is not registered with an RD [23], to make the two groups (registered with an RD or not) comparable on average. We considered patient demographic and pre-treatment (in the year 2011) outcome measures as variables in the propensity score matching analysis. The full list of propensity score matching variables and results is available in S2 Table. We applied the method proposed by Oster (2019) [24] to test the robustness of the results of the propensity score analysis to any bias arising from any unobservable characteristics that we could not control for and that may have explained individuals’ registration with an RD.

While propensity score matching controls for observable differences between those who did and did not register with an RD, the traditional difference-in-differences estimator compares the difference in the two groups, before and after the introduction of the policy. Therefore, it also controls for potential confounding from variables that are unobservable or unmeasured but do not vary over time, which propensity score matching - on its own - could not achieve [25]. The difference-in-differences method produces unbiased estimates of the policy impact if the trend over time between the two groups (registered or not with an RD) would have followed the same trajectory in the absence of the intervention. This ‘parallel trends’ assumption is violated if the two groups differed in ways that would change their trends or compositions over time. The use of propensity score matching makes the parallel trends assumption more feasible, as it reduces observable differences between PWT2D with and without an RD. Therefore, we kept only the sample of PWT2D with an RD that were matched to PWT2D without an RD and discarded unmatched observations. We then implemented the Callaway and Sant’Anna [26] difference-in-differences technique. This allowed us to account for the fact that individuals registered with an RD in different years, thereby overcoming a limitation of the traditional difference-in-differences estimator that assumes that all individuals registered with an RD at the same point in time. Additional technical detail on the statistical methods is available in S2 Text.

We analysed the effects of the programme in the short-term (until 2015, after which the programme eligibility changed) and medium-term (until 2018). We conducted a sensitivity analysis, which replicated the analyses including PWT2D who were ‘not yet’ enrolled with an RD in the comparison group (along with PWT2D who never enrolled with an RD) for patients already enrolled with an RD. We undertook statistical analyses in Stata 17 [27] and used the *psmatch 2* [28] command to estimate the propensity score model and the *csdid* [29] command to estimate the Callaway and Sant’Anna [26] difference-in-differences model.

The statistical analysis was complemented by five qualitative expert interviews with a total of seven interviewees representing GPs, the Ministry of Health, representative health sector federations and doctor federations in Luxembourg (see S3 Table). The objective of these expert interviews was to explore the implementation process of the RD programme in order to gain insight into the statistical results. During an initial exploratory phase and the elaboration of a sampling strategy, we identified the major representative associations, which identified several GPs, whom we contacted to take part in the study. All of the major representative health actors were interviewed as experts with a practical knowledge of the programme [30]. Only a handful of GPs agreed to be interviewed. However, this was amended by the fact that the qualitative data collection also benefitted from the fact that interviewees had multiple ‘hats’ (for example, one GP was a senior member of the national representative GP federation) and therefore had a more holistic perspective of the RD programme. We developed an interview guide based on our knowledge of the RD programme and the results of the statistical analysis (see S3 text). Informed consent was taken from participants to participate in the study. The interviews took place between May and June 2023 and lasted for up to one hour. The interviews were recorded, pseudonymised, transcribed, coded and analysed using thematic content analysis [31, 32] using the MAXQDA software [33].

## Results

**Fig. 1:**
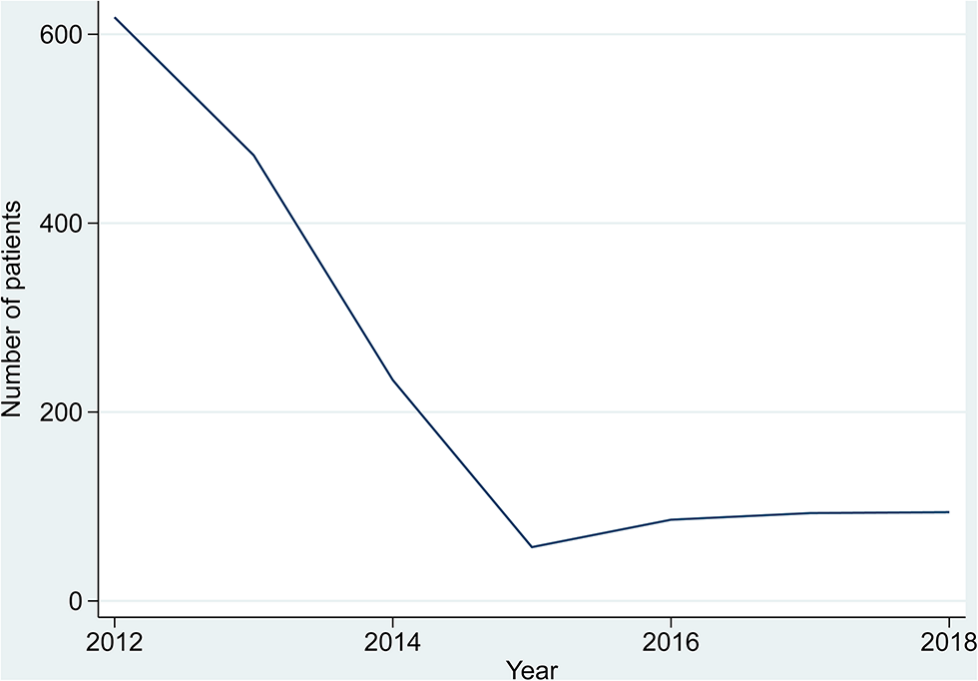
Number of people with type 2 diabetes who joined the RD programme, 2012–2018

Attrition due to patient deaths each year was relatively low, ranging from 1 to 3% (see S4 Table) and otherwise the panel was balanced. Figure 1 shows the number of PWT2D in the cohort who joined the programme each year.

Approximately 4% (618 patients) of PWT2D registered with an RD in 2012. The numbers who registered with an RD declined until 2015 and increased thereafter.

Table 1 presents the findings of the baseline (2011) characteristics of the full (unmatched) and matched samples. This shows that before matching, there were statistically significant differences at baseline (2011) between PWT2D who registered with an RD during the period 2012–2018 and PWT2D who did not register with an RD. A higher proportion of those registered with an RD were widowed. PWT2D who registered with an RD also had a higher average number of GP consultations, and a higher average number of repeat reimbursed prescribed medicines. However, these differences were no longer statistically significant after matching.

**Table 1:** Baseline (year 2011) characteristics of full (unmatched) and matched samples

	Full (unmatched) sample, *N* = 16,775		t-test		Matched sample, *N* = 2,694		t-test	
Variable	Registered with an RD	Not registered with an RD	T	*p* > t	Registered with an RD	Not registered with an RD	t	*p* > t
Sex (percentage of females)	45.46	44.03	1.03	0.302	45.46	47.07	-0.86	0.389
Age (average, years)	64.51	61.79	8.58	0.000	64.51	64.99	-1.17	0.241
*Civil Status (percentage of patients)*								
Married/Civil Partner	56.98	60.14	-2.30	0.021	56.98	55.10	1.02	0.310
Single	6.15	7.24	-1.52	0.128	6.15	6.15	0.00	1.000
Separated/Divorced	9.78	10.08	-0.36	0.717	9.78	9.64	0.13	0.900
Widow(er)	27.10	22.54	3.87	0.000	27.10	29.12	-1.21	0.228
*Utilisation (average number per patient per year)*								
GP consultations	5.13	3.73	14.41	0.000	5.13	5.03	0.67	0.501
GP home visits	0.21	0.19	0.99	0.322	0.21	0.20	0.46	0.645
Specialist consultations	4.01	3.99	0.18	0.858	4.01	4.12	-0.75	0.453
Cardiologist consultations	0.40	0.36	1.87	0.061	0.40	0.43	-0.95	0.341
*Quality of care process indicators (average number per patient per year)*								
Eye exam by an ophthalmologist	0.69	0.64	1.43	0.152	0.69	0.71	-0.44	0.658
Dental consultation	0.56	0.60	-1.52	0.129	0.56	0.55	0.13	0.899
HbA1c measurement	1.68	1.49	5.11	0.000	1.68	1.67	0.18	0.856
Complete cholesterol test	1.08	0.98	4.10	0.000	1.08	1.07	0.28	0.777
Kidney function (blood) test	1.30	1.16	4.51	0.000	1.30	1.27	0.57	0.567
Kidney function (urine) test	0.25	0.16	6.85	0.000	0.25	0.23	0.97	0.334
*Reimbursed prescribed medicines (average number per patient per year)*								
Total repeat (> 3 deliveries) reimbursed prescribed medicines	6.10	5.54	5.41	0.000	6.10	6.26	-1.20	0.230
Repeat ATC B – Blood and blood forming organs	0.40	0.35	3.40	0.001	0.40	0.42	-0.90	0.371
Repeat ATC C – Cardiovascular system	1.87	1.63	6.17	0.000	1.87	1.97	-1.76	0.079
Repeat ATC D - Dermatologicals	0.08	0.07	0.67	0.505	0.08	0.08	0.05	0.958
Repeat ATC N - Nervous system	0.82	0.72	2.68	0.007	0.82	0.88	-1.33	0.184
Repeat ACT S – Sensory organs	0.13	0.12	0.65	0.517	0.13	0.12	0.36	0.720

Table 2 shows the results of the difference-in-differences analysis.

**Table 2:** Difference-in-differences estimates, *p*-values and 95% confidence intervals

	Short-term (until 2015)				Medium-term (until 2018)			
Variable	Coefficient	*P*-value	95% Confidence Interval		Coefficient	*P*-value	95% Confidence Interval	
*Utilisation (number per patient per year)*								
GP consultations	0.43**^**	0.001	0.18	0.67	0.26**^**	0.026	0.03	0.50
GP home visits	0.01	0.715	-0.05	0.08	0.05**^**	0.174	-0.02	0.13
Specialist consultations	-0.23	0.066	-0.48	0.02	-0.23	0.050	-0.46	0.00
Cardiologist consultations	-0.01	0.848	-0.06	0.05	0.02	0.512	-0.04	0.07
*Quality of care process indicators (number per patient per year)*								
HbA1c test	0.08**^**	0.098	-0.01	0.17	0.09	0.030	0.01	0.18
Complete cholesterol test	-0.00**^**	0.948	-0.08	0.07	0.01	0.803	-0.06	0.08
Kidney function (blood) test	0.10	0.029	0.01	0.19	0.11	0.009	0.03	0.20
Kidney function (urine) test	0.06**^**	0.009	0.02	0.11	0.06	0.008	0.02	0.10
Eye exam by an ophthalmologist	0.01	0.756	-0.07	0.10	0.01	0.905	-0.08	0.09
Dental consultation	0.03	0.295	-0.03	0.10	0.02	0.583	-0.04	0.07
*Reimbursed prescribed medicines (number per patient per year)*								
Total repeat (> 3 deliveries) reimbursed prescribed medicines	0.19	0.022	0.03	0.36	0.18	0.032	0.02	0.34
Repeat ATC B – Blood and blood forming organs	0.02	0.265	-0.01	0.05	0.02	0.198	-0.01	0.05
Repeat ATC C – Cardiovascular system	0.07**^**	0.038	0.00	0.13	0.08	0.017	0.01	0.15
Repeat ATC D - Dermatologicals	-0.01	0.569	-0.03	0.02	-0.02	0.182	-0.04	0.01
Repeat ATC N - Nervous system	0.05	0.096	-0.01	0.11	0.05	0.093	-0.01	0.11
Repeat ACT S – Sensory organs	0.00	0.873	-0.02	0.02	-0.01	0.637	-0.03	0.02

**^**The difference-in- differences assumption of parallel trends does not hold.

### Use of general practitioner (GP) and specialist care

We were unable to make inferences for GP consultations and GP home visits (medium-term) as the parallel trends assumption did not hold (denoted by ^ in Table 2). There were statistically insignificant increases in GP home visits in the short-term (0.01 (95% CI:-0.05 to 0.08), and statistically insignificant decreases in specialist consultations in the short- (-0.23 (95% CI:-0.48 to 0.02)) and medium-term (-0.23 (95% CI:-0.46 to 0.00)). Cardiologist consultations decreased in the short-term (-0.01 (95% CI:-0.06 to 0.05) and increased in the medium-term (0.02 (95% CI:-0.04 to 0.07) but these were also not statistically significant.

### Quality of care process indicators

The parallel trends assumption did not hold for the following measures in the short-term (denoted by ^ in Table 2): HbA1c test, complete cholesterol test, and kidney function (urine) test, meaning that we could not make inferences using these estimates. There was a statistically significant increase in the number of HbA1c tests (0.09 (95% CI: 0.01 to 0.18)) in the medium-term, which represented an increase of 5.5% of the baseline value (see Table 1) of the group of PWT2D registered with an RD. There was also an increase in the number of kidney function (blood) tests in the short- (0.10 (95% CI: 0.01 to 0.19)) and medium-term (0.11 (95% CI: 0.03 to 0.20)), which represented an increase of 7.8% and 8.8% of the baseline value (see Table 1) of the group of PWT2D registered with an RD. There was a statistically significant increase in the number of kidney function (urine) tests (0.06 (95% CI: 0.02 to 0.10)) in the medium-term, which represented an increase of 23.6% of the baseline value (see Table 1) of the group of PWT2D registered with an RD. There were statistically insignificant increases in the number of: complete cholesterol tests (medium-term) (0.01 (95% CI:-0.06 to 0.08)), eye exams by an ophthalmologist in the short- (0.01 (95% CI:-0.07 to 0.10)) and medium-term (0.01 (95% CI:-0.08 to 0.09)), and dental consultations in the short- (0.03 (95% CI:-0.03 to 0.10)) and medium-term (0.02 (95% CI:-0.04 to 0.07)).

### Reimbursed prescribed medicines

The parallel trends assumption did not hold for the number of repeat prescribed cardiovascular system medicines (short-term), meaning that we could not make inferences using these estimates (denoted by ^ in Table 2). The total number of repeat prescribed medicines increased by 0.19 (95% CI: 0.03 to 0.36) in the short-term and 0.18 (95% CI: 0.02 to 0.34) in the medium-term, an increase of 3% of the baseline value (see Table 1) of the group of PWT2D registered with an RD. There was also an increase in the number of repeat prescribed cardiovascular system medicines (0.08 (95% CI: 0.01 to 0.15)) in the medium-term, which represented an increase of 4.3% of the baseline value (see Table 1) of the group of PWT2D registered with an RD.

There were statistically insignificant increases in the number of repeat prescribed medicines for blood and blood forming organs (ATC B) in the short- (0.02 (95% CI:-0.01 to 0.05)) and medium-term (0.02 (95% CI:-0.01 to 0.05)); and the nervous system (ATC N in the short- (0.05 (95% CI:-0.01 to 0.11)) and medium-term (0.05 (95% CI:-0.01 to 0.11)), and statistically insignificant decreases in the number of repeat prescribed medicines for: dermatologicals (ATC D) in the short- (-0.01 (95% CI:-0.03 to 0.02)) and medium-term (-0.02 (95% CI:-0.04 to 0.01)); and the sensory organs in the medium-term (-0.01 (95% CI:-0.03 to 0.02)).

### Additional outcomes

Regarding the additional outcomes considered (S5 Table), the parallel trends assumption did not hold for the variables measuring GP costs, meaning that we could not make inferences based on these estimates. We did not find evidence of an impact of the RD policy on the other additional outcomes.

### Sensitivity analyses

Our application of Oster (2019) [24] revealed that the influence of patients’ unobservable characteristics on the estimates were less important than that of the included control variables in explaining registration with an RD (results available on request). This implies that our results were robust to the presence of unobservable or omitted characteristics in the analysis.

The results of the sensitivity analysis that expanded our sample to consider the additional 6,769 patients with deliveries of insulin as well as oral medication are available in S6 Table. The results were somewhat sensitive to the increased heterogeneity and size of the sample. The parallel trends assumption did not hold for the variables measuring the number of: HbA1c tests (medium-term), kidney function (blood) tests, and cardiovascular system medicines (medium-term) meaning that inferences could no longer be made on these estimates. However, the parallel trends assumption was met for the number of kidney function (urine) tests in the short-term, which showed an increase (0.07, 95% CI: 0.03 to 0.11), representing 28% of the baseline value (see Table 1) of the group of PWT2D registered with an RD. There was also a statistically significant increase in the number of dental consultations (0.06, 95% CI: 0.00 to 0.11) in the short-term, representing 11% of the baseline value (Table 1) of the group of PWT2D registered with an RD. The results for the number of kidney function (urine) tests in the short-term (0.07, 95% CI: 0.03 to 0.11) and the total number of repeat prescribed medicines in the short- (0.19 (95% CI: 0.04 to 0.35)) and medium-term (0.20 (95% CI: 0.05 to 0.36)) were very similar to the main analysis.

The results of the analysis that also included PWT2D who had not yet enrolled with an RD in the comparison group for people already enrolled with an RD (S7 Table) confirmed the main results. This analysis also revealed statistically significant decreases in specialist consultations in the short- (-0.28, 95% CI: -0.51 to -0.04) and medium- (-0.26, 95% CI: -0.49 to -0.03) terms. These represented a decrease of 6.9% and 6.5% of the baseline values (see Table 1) of the group of PWT2D registered with an RD. While the parallel trends assumption was fulfilled for the variable measuring the number of repeat prescribed cardiovascular system medicines (short-term), the effect was not statistically significant.

## Discussion

Many European countries have sought to strengthen primary care through patient registration with a primary care provider. Registration is a vehicle to improve continuity and coordination of care, in order to address the needs of patients with chronic illness. We contribute to the limited literature on patient registration, by evaluating the effects of a voluntary primary care patient registration programme in Luxembourg, a health care system with free choice of primary and specialist doctor, and no primary care gatekeeping.

The RD policy was associated with increases in quality of care process indicators, most notably for the number of kidney function (urine) tests in the medium term, which increased by 24% compared to the baseline value of 0.25 of a test among PWT2D registered with an RD. This implies that the programme was effective in increasing the prescribing of a test with low baseline levels but had limited effect for other indicators that met the threshold recommended by the clinical guidelines. The increases in the number of HbA1c tests and kidney function (blood) tests were relatively small and were sensitive to the expansion of the sample to PWT2D who were also prescribed insulin. We also found evidence of a miniscule increase in the number of repeat reimbursed prescribed medicines, which would not necessarily raise concerns about polypharmacy. Moreover, the increase in reimbursed prescribed cardiovascular system medicines could indicate that doctors are identifying other chronic conditions or comorbidities. There was a reduction in specialist consultations in the short- and medium-term when we compared PWT2D who enrolled in the programme in earlier years to those who enrolled in later years (not yet enrolled) together with those who never enrolled. The enlarged control group likely increased the precision of the estimates. We also found an increase in the number of dental consultations in the short-term when we increased the sample to include PWT2D who also had deliveries of insulin. The programme did not have a statistically significant effect on GP home visits or cardiologist consultations.

There was a relatively low registration with an RD among PWT2D. Some interviewees indicated that the RD programme was not well known, implying a need to promote the programme among doctors and patients. While patient registration is voluntary in many countries, there are often incentives for patients to register including lower user charges and easier access to GPs and specialist doctors [2]. In Luxembourg, the RD programme could be reformed to include an additional incentive for patients, such as a reduction in or even an abolishment of co-payments for specific services, which could encourage more patients to register with an RD. Some expert interviewees suggested that an implicit benefit for patients was an activated electronic health record, which could prove particularly advantageous in the event of a health emergency, as health professionals with access to the record would have immediate access to information on patients’ health conditions and treatment. Therefore, patients could be informed and encouraged to authorise access to their electronic health record for health professionals beyond the RD, in order to increase its benefit, especially when urgent care is required. While GPs receive additional payment for participation in the RD programme, the GP interviewees discussed a series of issues that could be regarded as potential disincentives for doctors to participate, including the lack of an integrated IT system and technical issues, leading to additional effort in terms of time and work.

A strength of our study was the use of a rich administrative dataset, which allowed us to investigate several relevant outcomes and to assess the short- and medium-term effects of the policy. In the context of observational studies, our use of propensity score matching combined with difference-in-differences analysis ensured an unbiased estimate of the RD programme. We also applied a recent methodological innovation in difference-in-differences studies, to account for PWT2D registering with an RD in different years. Therefore, we also contribute to the limited literature [34–38] that have applied these innovative methods to empirically evaluate health policy reforms. The use of the National Health Insurance Fund data meant that we could only consider patients with *treated* type 2 diabetes, meaning that we excluded patients with untreated diabetes. This could be a non-negligible group, as it is reported that approximately one-third of PWT2D are commonly undiagnosed [12, 15]. The interpretation of our results was limited by the absence of clinical data. For example, an increased number and frequency of HbA1c tests may be required, if HbA1c is uncontrolled or above the target range. We also lacked information on the clinical outcomes of patients. While many tests were performed on the same data, which is associated with an increased risk of Type 1 errors, we have reason to believe that this is not a cause for concern as (i) Austin (2009) [39] has shown that the use of a propensity-score matched sample tended to result in Type 1 error rates that were closer to the advertised level compared to the use of an unmatched sample, and (ii) there were statistically significant results (and the parallel trends assumption was met) for only five of the sixteen (in the short- and medium-term) outcomes analysed.

Although our study evaluated the effects of a policy focused on GP registration, for comparison purposes we can consider related literature on primary care reforms in neighbouring countries that included an element of GP registration. Studies of reforms including voluntary patient registration in France and Germany demonstrated that they achieved some success in meeting their objectives in terms of reducing the number of specialists and different GPs visited [40] and improved coordination of care [41]. In their evaluation of a ‘GP-centred health care’ programme in Germany, Freytag et al. (2016) [41] also found an increase in GP consultations and home visits as well as an increase in specialist consultations. In their evaluation of the médecin traitant reform in France, Dumontet et al. (2017) [40] reported a decrease in visits to specialists following the reform, similar to our findings. However, previous studies were limited by a lack of a plausible comparison group [40] and a short (18-month) follow-up period [41].

## Conclusions

Patient registration with a primary care provider has the potential to improve care coordination and strengthen primary care. We add to the limited evidence base on the effects of patient registration within countries. Our results show that a voluntary patient registration programme that does require or incentivise registered patients to obtain a referral to specialist care, had a limited effect on care quality indicators and reimbursed prescribed medicines for PWT2D. Future research could explore the impact of the programme on other chronic conditions and consider heterogeneity in the effects of the policy, for example, whether there are different effects for newly diagnosed diabetes patients or patients with multimorbidity. Another important avenue for future research is the linkage of the National Health Fund data with other datasets that would facilitate the inclusion of data on socio-economic characteristics and clinical and patient-reported outcome measures.

### Electronic supplementary material

Below is the link to the electronic supplementary material.

Supplementary Material 1

## Data Availability

The National Health Fund data used for this study is not publicly available.

## Abbreviations

GP: general practitioner
RD: Referring Doctor
PWT2D: people with type 2 diabetes on oral medication
