# Supplementary material for: The impact of patient registration on utilisation and quality of care: a propensity score matching and staggered difference-in-differences analysis of a cohort of 16,775 people with type 2 diabetes
Source: BMC Prim Care. 2024 Jul 12;25:254. doi: 10.1186/s12875-024-02505-2 (PMC11245844; doi:10.1186/s12875-024-02505-2)
Supplement: Supplementary file 1 — Supplementary Material 1 [file 12875_2024_2505_MOESM1_ESM.docx]

**Article title: The impact of patient registration on utilisation and quality of care: a propensity score matching and staggered difference-in-differences analysis of a cohort of 16,775 people with type 2 diabetes**

**Authors’ information:**

Valerie Moran^1, 2^*, Michela Bia^3^, Patrick Thill^3^, Marc Suhrcke^1, 2^, Ellen Nolte^4^, Eric Burlot^5^, Guy Fagherazzi^6^

1 Socio-Economic and Environmental Health and Health Services Research Group, Department of Precision Health, Luxembourg Institute of Health, Luxembourg (corresponding author: valerie.moran@lih.lu)

2 Socio-Economic and Environmental Health and Health Services Research Group, Living Conditions Department, Luxembourg Institute of Socio-Economic Research, Luxembourg (corresponding author: valerie.moran@liser.lu)

3 Labour Market Department, Luxembourg Institute of Socio-Economic Research, Luxembourg

4 Department of Health Services Research and Policy, London School of Hygiene and Tropical Medicine, United Kingdom

5 Nomenclature, Conventions, Analysis and Forecasting Department, National Health Insurance Fund, Luxembourg

6 Deep Digital Phenotyping Research Unit, Department of Precision Health, Luxembourg Institute of Health, Luxembourg

**Supplementary material**

**S1 Text. DIABECOLUX algorithm developed by Renard et al. (2011)**

- Criterion 1: 3 deliveries (or more) of A10 per year, for 2 years or more;
- Criterion 2: 3 deliveries of A10 (or more) for 1 year AND 2 deliveries per year, for 2 years or more;
- Criterion 3: 2 deliveries of A10 per year, for 3 years or more - to consider patients often abroad;
- Criterion 4: 3 deliveries of A10 (or more) for the year of Death (X), OR the year X-1, OR 2017, OR 2018 - to consider right truncation and include the incident cases of the last years of the period.

The application of the DIABECOLUX algorithm identified 36,289 patients treated for diabetes during the period 2010-2018. In order to follow a defined cohort over the period of the policy, we restricted the sample to 25,259 patients with at least two A10 deliveries before or during 2012. We excluded pregnant women (n=151) to exclude gestational diabetes. This resulted in a cohort of 25,108 patients. We then identified if patients had Type 1 or Type 2 diabetes based on whether they had deliveries of only insulin (ATC code A10A) (n=1,564, 6%) or only oral medications (ATC code A10B), (n=16,775, 67%). Just over one-quarter of the cohort (n=6,769, 27%) had received deliveries of both insulin and oral treatment.

**S1 Table. Variables description**

| **Variable** | **Description** |
| --- | --- |
| *Demographic variables* | |
| Sex | Binary variable equal to zero for male and one for female |
| Age | Number of years, calculated by subtracting the year of birth from each year of the study (2010-2018) |
| *Civil status* | |
| Married/civil partnership | Binary variable equal to one if married or in a civil partnership and equal to zero if single, separated/divorced or widowed |
| Single | Binary variable equal to one if single and equal to zero if married/civil partnership, separated/divorced, widowed |
| Separate/divorced | Binary variable equal to one if separated or divorced and equal to zero if single, married/civil partnership or widowed |
| Widow(er) | Binary variable widow(er) (equal to one if widow(er) and equal to zero if married/civil partnership, single or separated/divorced) |
| Canton of residence | A binary variable for each canton with an additional variable to differentiate between Luxembourg city and countryside. |
| *Utilisation, number per year* | |
| GP consultation | Consultations with a GP |
| Specialist consultation | Consultations with a specialist doctor (except cardiologists) |
| Cardiologist consultation | Consultations with a cardiologist |
| GP home visits | Visits of a GP to a patient (at the patient’s home, place of residence, or in a hospital) |
| Urgent or out-of-hours consultations* | Consultations that were urgent or took place between 8pm and 7am on a weekday, or on a Sunday or public holiday |
| Urgent or out-of-hours visits* | Number of visits (to a patient’s residence or in a hospital) that were urgent or took place between 8pm and 7am on a weekday, or on a Sunday or public holiday |
| Inpatient admissions* | Inpatient (including psychiatric) admissions |
| Day admissions* | Day hospital admissions |
| *Costs, per year* |  |
| GP costs* | Total GP costs, Euros inflated to 2018 prices^§^ |
| Specialist costs * | Total specialist costs, Euros inflated to 2018 prices^§^ |
| *Care quality indicators, number per year (continuous variables)* | |
| Eye exams by an ophthalmologist | Examination of the eye fundus by an ophthalmologist |
| Dental consultation | Dental consultation |
| HbA1c test | HbA1c blood test |
| Complete cholesterol test | A patient is considered to have received a complete cholesterol test if they received at least three of the following four laboratory tests on the same date: total cholesterol, high-density lipoprotein (HDL) cholesterol, low-density lipoprotein (LDL) cholesterol, triglycerides |
| Kidney function (blood) test | Creatinine blood test (used to estimate the glomerular filtration rate) |
| Kidney function (urine) test | One albumin urine test and one creatinine urine test on the same date. These tests are used to calculate the albumin-to-creatinine ratio. We cannot calculate the ratio but assume that the presence of both tests on the same date enables this calculation. |
| *Reimbursed prescribed medicines, number per year* | |
| Total repeat (>3 deliveries) reimbursed prescribed medicines | Reimbursed prescribed medicines with at least three deliveries per year |
| Repeat ATC B prescribed medicines - Blood and blood forming organs | ATC B (Blood and blood forming organs) reimbursed prescribed medicines with at least three deliveries per year |
| Repeat ATC C – Cardiovascular system | ATC C (Cardiovascular system) reimbursed prescribed medicines with at least three deliveries per year |
| Repeat ATC D - Dermatologicals | ATC D (Dermatologicals) reimbursed prescribed medicines with at least three deliveries per year |
| Repeat ATC N - Nervous system | ATC N (Nervous system) reimbursed prescribed medicines with at least three deliveries per year |
| Repeat ATC S - Sensory organs | ATC S (Sensory organs) reimbursed prescribed medicines with at least three deliveries per year |
| *Continuity of care* |  |
| Number of different GPs reimbursed for a patient* | Number of different GPs reimbursed for a patient per year |

*Additional outcomes: results reported in S5 Table.

§ We inflated costs to 2018 prices using the Consumer Price Index for health published by the Luxembourgish National Institute of Statistics and Economic Studies <https://statistiques.public.lu/en/themes/economie-finances/dossier-indice-prix-inflation.html>

**S2 Table. Propensity score matching variables and results**

|  | **Unmatched (n=16,775)** | **Mean** | |  |  | **t-test** | |
| --- | --- | --- | --- | --- | --- | --- | --- |
| **Variable** | **Matched (n=2,694)** | **Treated** | **Control** | **% bias** | **% reduction bias** | **t** | **p>\|t\|** |
| Sex | U | 0.45 | 0.44 | 2.9 |  | 1.03 | 0.302 |
|  | M | 0.45 | 0.47 | -3.2 | -11.9 | -0.86 | 0.389 |
| Age | U | 64.51 | 61.79 | 24.4 |  | 8.58 | 0.000 |
|  | M | 64.51 | 64.99 | -4.3 | 82.4 | -1.17 | 0.241 |
| *Civil status* | | | | | | | |
| Married/civil partnership | U | 0.57 | 0.60 | -6.4 |  | -2.30 | 0.021 |
|  | M | 0.57 | 0.55 | 3.8 | 40.3 | 1.02 | 0.310 |
| Single | U | 0.06 | 0.07 | -4.4 |  | -1.52 | 0.128 |
|  | M | 0.06 | 0.06 | 0 | 100 | 0.00 | 1.000 |
| Separated/divorced | U | 0.10 | 0.10 | -1 |  | -0.36 | 0.717 |
|  | M | 0.10 | 0.10 | 0.5 | 54.1 | 0.13 | 0.900 |
| Widow(er) | U | 0.27 | 0.23 | 10.6 |  | 3.87 | 0.000 |
|  | M | 0.27 | 0.29 | -4.7 | 55.6 | -1.21 | 0.228 |
| *Canton of residence* | | | | | | | |
| Capellen | U | 0.11 | 0.07 | 12 |  | 4.62 | 0.000 |
|  | M | 0.11 | 0.10 | 0.7 | 93.9 | 0.18 | 0.855 |
| Clervaux | U | 0.08 | 0.02 | 24.3 |  | 11.14 | 0.000 |
|  | M | 0.08 | 0.07 | 2.2 | 90.7 | 0.50 | 0.618 |
| Diekirch | U | 0.05 | 0.07 | -9.4 |  | -3.13 | 0.002 |
|  | M | 0.05 | 0.04 | 2.1 | 77.3 | 0.64 | 0.519 |
| Echternach | U | 0.03 | 0.03 | 1.2 |  | 0.44 | 0.658 |
|  | M | 0.03 | 0.03 | 0.4 | 68.1 | 0.10 | 0.918 |
| Esch sur Alzette | U | 0.40 | 0.36 | 8.5 |  | 3.05 | 0.002 |
|  | M | 0.40 | 0.41 | -1.6 | 81.3 | -0.42 | 0.676 |
| Grevenmacher | U | 0.04 | 0.05 | -3.7 |  | -1.29 | 0.196 |
|  | M | 0.04 | 0.05 | -3 | 19.9 | -0.80 | 0.421 |
| Luxembourg (countryside) | U | 0.06 | 0.10 | -13.1 |  | -4.34 | 0.000 |
|  | M | 0.06 | 0.06 | -0.3 | 98 | -0.08 | 0.938 |
| Luxembourg (city) | U | 0.08 | 0.12 | -16.2 |  | -5.36 | 0.000 |
|  | M | 0.08 | 0.06 | 3.5 | 78.4 | 1.10 | 0.273 |
| Mersch | U | 0.08 | 0.05 | 9.8 |  | 3.77 | 0.000 |
|  | M | 0.08 | 0.09 | -3.4 | 65.2 | -0.82 | 0.411 |
| Redange | U | 0.03 | 0.03 | 0.9 |  | 0.32 | 0.748 |
|  | M | 0.03 | 0.03 | 1.2 | -30.9 | 0.31 | 0.754 |
| Remich | U | 0.02 | 0.05 | -17.3 |  | -5.30 | 0.000 |
|  | M | 0.02 | 0.02 | 1.2 | 93.2 | 0.44 | 0.659 |
| Vianden | U | 0.01 | 0.01 | -3.3 |  | -1.12 | 0.263 |
|  | M | 0.01 | 0.01 | 0 | 100 | 0.00 | 1.000 |
| Wiltz | U | 0.02 | 0.03 | -5.7 |  | -1.90 | 0.057 |
|  | M | 0.02 | 0.03 | -2.6 | 53.6 | -0.74 | 0.462 |
| *Utilisation* | | | | | | | |
| GP costs | U | 227.80 | 172.65 | 33 |  | 12.00 | 0.000 |
|  | M | 227.80 | 221.24 | 3.9 | 88.1 | 0.96 | 0.339 |
| Specialist costs | U | 838.72 | 792.06 | 3.7 |  | 1.34 | 0.181 |
|  | M | 838.72 | 848.43 | -0.8 | 79.2 | -0.21 | 0.835 |
| GP consultation | U | 5.13 | 3.73 | 38.3 |  | 14.41 | 0.000 |
|  | M | 5.13 | 5.03 | 2.9 | 92.6 | 0.67 | 0.501 |
| GP home visits | U | 0.21 | 0.19 | 2.6 |  | 0.99 | 0.322 |
|  | M | 0.21 | 0.20 | 1.7 | 34.4 | 0.46 | 0.645 |
| Specialist Consultation | U | 4.01 | 3.99 | 0.5 |  | 0.18 | 0.858 |
|  | M | 4.01 | 4.12 | -2.8 | -446.7 | -0.75 | 0.453 |
| Cardiologist consultation | U | 0.40 | 0.36 | 5.1 |  | 1.87 | 0.061 |
|  | M | 0.40 | 0.43 | -3.7 | 27.3 | -0.95 | 0.341 |
| Specialist home visits | U | 0.04 | 0.04 | 0 |  | -0.01 | 0.990 |
|  | M | 0.04 | 0.05 | -0.5 | -1106.3 | -0.10 | 0.919 |
| Inpatient admissions | U | 0.26 | 0.24 | 4.4 |  | 1.61 | 0.108 |
|  | M | 0.26 | 0.26 | 0.6 | 87.4 | 0.14 | 0.886 |
| Day admissions | U | 0.13 | 0.12 | 2.1 |  | 0.74 | 0.461 |
|  | M | 0.13 | 0.14 | -2.7 | -31.6 | -0.69 | 0.491 |
| Psychiatric inpatient admissions | U | 0.03 | 0.04 | -0.6 |  | -0.18 | 0.856 |
|  | M | 0.03 | 0.05 | -1.9 | -225 | -0.48 | 0.635 |
| Rehabilitation admissions | U | 0.01 | 0.00 | 3 |  | 1.16 | 0.245 |
|  | M | 0.01 | 0.01 | 1 | 67.4 | 0.24 | 0.808 |
| Urgent or out-of-hours consultations | U | 0.42 | 0.43 | -0.8 |  | -0.29 | 0.768 |
|  | M | 0.42 | 0.43 | -1.1 | -32.5 | -0.29 | 0.772 |
| Urgent or out-of-hours visits | U | 0.13 | 0.13 | -1 |  | -0.35 | 0.727 |
|  | M | 0.13 | 0.12 | 2 | -91.6 | 0.58 | 0.559 |
| *Care quality indicators* | | | | | | | |
| Eye exams by an ophthalmologist | U | 0.69 | 0.64 | 4 |  | 1.43 | 0.152 |
|  | M | 0.69 | 0.71 | -1.7 | 57.2 | -0.44 | 0.658 |
| Dental consultation | U | 0.56 | 0.60 | -4.4 |  | -1.52 | 0.129 |
|  | M | 0.56 | 0.55 | 0.5 | 89.4 | 0.13 | 0.899 |
| HbA1c test | U | 1.68 | 1.49 | 14.5 |  | 5.11 | 0.000 |
|  | M | 1.68 | 1.67 | 0.7 | 95.3 | 0.18 | 0.856 |
| Complete cholesterol test | U | 1.08 | 0.98 | 11.4 |  | 4.10 | 0.000 |
|  | M | 1.08 | 1.07 | 1.1 | 90.6 | 0.28 | 0.777 |
| Kidney function (blood) test | U | 1.30 | 1.16 | 12.1 |  | 4.51 | 0.000 |
|  | M | 1.30 | 1.27 | 2.2 | 81.9 | 0.57 | 0.567 |
| Kidney function (urine) test | U | 0.25 | 0.16 | 16.7 |  | 6.85 | 0.000 |
|  | M | 0.25 | 0.23 | 3.9 | 76.4 | 0.97 | 0.334 |
| *Lab tests* | | | | | | | |
| Total number of blood tests | U | 9.97 | 8.88 | 16.2 |  | 5.78 | 0.000 |
|  | M | 9.97 | 9.99 | -0.4 | 97.4 | -0.11 | 0.910 |
| Total number of urine tests | U | 1.74 | 1.30 | 24 |  | 9.54 | 0.000 |
|  | M | 1.74 | 1.68 | 3.5 | 85.2 | 0.88 | 0.378 |
| *Reimbursed prescribed medicines* | | | | | | | |
| Total reimbursed prescribed medicines | U | 13.41 | 12.48 | 13.2 |  | 4.72 | 0.000 |
|  | M | 13.41 | 13.46 | -0.6 | 95.3 | -0.17 | 0.865 |
| Total repeat (>3 deliveries) reimbursed prescribed medicines | U | 6.10 | 5.54 | 15.2 |  | 5.41 | 0.000 |
|  | M | 6.10 | 6.26 | -4.4 | 70.9 | -1.20 | 0.230 |
| Repeat ATC B - Blood and blood forming organs | U | 0.40 | 0.35 | 9.4 |  | 3.40 | 0.001 |
|  | M | 0.40 | 0.42 | -3.5 | 62.5 | -0.90 | 0.371 |
| Number of ATC C - Cardiovascular system | U | 1.87 | 1.63 | 17 |  | 6.17 | 0.000 |
|  | M | 1.87 | 1.97 | -6.7 | 60.4 | -1.76 | 0.079 |
| Number of ATC D - Dermtologicals | U | 0.08 | 0.07 | 1.8 |  | 0.67 | 0.505 |
|  | M | 0.08 | 0.08 | 0.2 | 89 | 0.05 | 0.958 |
| Number of ATC N – Nervous system | U | 0.82 | 0.72 | 7.5 |  | 2.68 | 0.007 |
|  | M | 0.82 | 0.88 | -5.2 | 30.5 | -1.33 | 0.184 |
| Number of ATC S – Sensory organs | U | 0.13 | 0.12 | 1.8 |  | 0.65 | 0.517 |
|  | M | 0.13 | 0.12 | 1.3 | 26.2 | 0.36 | 0.720 |
| *Continuity of care* | | | | | | | |
| Number of different GPs reimbursed for a patient | U | 1.19 | 1.08 | 16.2 |  | 5.39 | 0.000 |
|  | M | 1.19 | 1.21 | -2.3 | 85.7 | -0.61 | 0.545 |

**S2 Text. Technical description of propensity score matching, difference-in-differences and Oster methods**

Formally, we consider a set of N individuals, and denote each of them by subscript i: i = 1, ...,N. For each individual i,…,N we observe a vector of covariates prior to registering with an RD, Xi and the value of the outcome variable associated with registration with an RD, Yi(1) for being registered with an RD, Yi(0) for not registering with an RD. Let p(X) be the probability of being exposed to the RD policy, given the set of covariates X: p(X) = Pr(D = 1|X = x).

Following the application of the propensity score matching, it is important to assess the quality of the matched samples in terms of covariate balance (the similarity of the distributions of the covariates in the matched groups of PWT2D registered or not with an RD).

Rubin (2001) recommends that the absolute standardised difference of means between the treated and control groups should be less than 25% and the variance ratios should be between 0.5 and 2. Nearest neighbour matching results in values of 17.9% and 0.87 respectively, implying that there is good covariate balance between the two groups.

Following Callaway and Sant’Anna (2021), we reformulate the corresponding outcome variable(s) and registration status as${(Yi,1, Yi,2...Yi,T ;Di,1,Di,2...Di,T )}_{i=1}^{N}$, where D*_i,t_* = 1 if unit *i* is registered with an RD in period *t*, and *0* otherwise. Now, let *G_i,g_* be equal 1 if unit *i* is first registered at time *g*, and 0 otherwise (“Treatment starting-time / Cohort dummies” ), while C = 1 is a group of PWT2D who never register with an RD. We also allow for PWT2D to register with an RD in different years, which is formalized as follows:

*D_i ,t_ = 1 → D_i ,t+1_ = 1 for t = 1, 2, ...,T.*

The parameter of interest is defined as:

*ATT(g, t) = E[Y_t_(g) − Yt(0)|G_g_ = 1]* for *t ≥ g*

which represents the average effect of registration with an RD for the group of PWT2D who first register at time period *g*, in calendar time *t.* Under the difference-in-differences econometric strategy, the identification strategy requires that the assumption on the conditional parallel trends based on the ed group of PWT2D who never register with an RD is satisfied, namely:

*E[Y_t_(0) – Y_t-1_(0)|Gg = 1] = E[Yt(0) – Y_t-1_(0)|C = 1],* for each *t ∈ 2, ...,T, g ∈ G* such that *t ≥ g.*

The Oster method evaluates omitted variable bias under the assumption that the relationship between treatment and unobservable characteristics can be recovered from the relationship between treatment and observable characteristics. Oster (2019) develops a consistent, closed-form estimator for omitted variable bias, which leads to a consistent estimate of the bias-adjusted treatment effects. The underlying assumptions are that the R^2^, derived from an hypothetical regression of the outcome on treatment and both observed and unobserved controls, is equal to 1 and that there is proportional selection on observed and unobserved variables (Altonji, et al. 2005). Using this methodology, we also compute the degree of selection on unobservable characteristics, in relation to the observables, which drives the estimated effect towards zero.

**References:**

Altonji J.G., Elder T.E., Taber, C.R., Selection on Observed and Unobserved Variables: Assessing the Effectiveness of Catholic Schools. Journal of Political Economy. 2005;113(1):151-84.

Callaway B, Sant’Anna PHC. Difference-in-Differences with multiple time periods. Journal of Econometrics. 2021;225(2):200-30.

Oster E. Unobservable Selection and Coefficient Stability: Theory and Evidence. Journal of Business & Economic Statistics. 2019;37(2):187-204.

Rubin DB. Using Propensity Scores to Help Design Observational Studies: Application to the Tobacco Litigation. Health Services and Outcomes Research Methodology. 2001;2(3):169-88.

**S3 Table. List of expert interviews**

| **Total of interviews** | **Interviews** | **Level of experience** | **Other affiliation** | **Location** | **Research focus** |
| --- | --- | --- | --- | --- | --- |
| 5 interviews with a total of 7 experts | 1 GP | Senior | None | Central | GP and patient experience |
|  | 1 GP | Junior | CMG  AAMD | North | GP and patient experience, experience of the CMG |
|  | 3 *Patientenvertriedung (Patient association)* | Senior | LCGB  OGBL | N/A | Association agenda and patient experience |
|  | 1 Ministry of Health | Senior | None | Central | Health policy, the history of the legislation, GP experience |

**S3 Text. Interview guide**

1. **Introduction**

The interviewer addresses terms of confidentiality and anonymity (check if statement is signed by interviewee).

The interviewer asks if he can record the interview electronically.

The interviewer explains that the material is stored in a secure server and destroyed at the end of the project.

The interviewer conducts a personal presentation

The interviewer explains the purpose of the interview and presents the project.

The interviewer explains the format and the length of the interview (1 to 1.5 hours max).

The interviewer asks a presentation of the interviewee: function in the institution/association, date of entry in function.

The interviewer asks if interviewee has any questions before the start of the interview.

The interviewer asks the interviewee to share the story of the interviewees’ professional journey and what has brought the interviewee to where the interviewee is today (**warming-up question**)

1. **Professional experience with the MR (All interviewees)**

When and where was your first encounter with the MR programme?

Can you describe briefly how the MR scheme works in practice according to your experience? What general feed-back do you receive from patients?

What have been the positive and negative aspects (problems) of the programme according to your daily experience? Examples?

What was the motivation for introducing the MR programme? Was this motivation successful and efficient? Was there any opposition to the introduction?

Did you consider the design and implementation of similar programmes (e.g. in France or Germany)? Did the experience of other countries influence the design of the MR programme?

Was your association involved in the initial discussions, debates or negotiations, polcymaking? In the implementation process? Can you describe the implementation process?

If yes, to what extent and what did your association contribute? Examples? If yes, can you describe the negotiation process? Criticisms?

Was the MR scheme discussed in collective bargaining arenas? (“Gesondheetsdesch”)

How was the situation of primary care before the MR reform? What was better?

More broadly: what is the role of primary care in the health system in Luxembourg compared to other countries? Examples.

How do you characterise the role of the MR programme within the overall national health system?

What is the importance of digital tools in the MR programme?

How has the MR programme changed over the years in your opinion? Examples.

Did the change in eligibility improve the programme? If not, why?

Do you think the programme could be further improved and if so, how?

Do you think a stronger role for primary care doctors in referring patients to specialist care (i.e. gate-keeping) is required?

What have been motivations of GPs to participate in the MR programme (i.e. financial payment) according to your experience? What are the motivations for patients?

How do doctors and patients obtain information about the programme or know how it exists?

What is the relation between the MR and other actors such as the Ministry or AMMD? Can you describe the communication between the actors? Is there an active promotion of the programme? Examples.

What could be the motivations (for doctors and/or patients) not to participate or leave? Is there too much administrative work involved? Could this be a negative aspect of the programme? Examples?

In general, there is low participation by patients in the programme – what are the reasons for this in your opinion?

Are there differences in participation by GPs in the programme the country (if yes, can probe if this could be linked to patient characteristics e.g. age, health status or socio-economic)?

1. **Professional experiences MR (GP section only)**

When and where was your first encounter with the MR programme?

What have been your motivations to participate and sign up in the MR programme (i.e. financial payment)?

What are the motivations for patients to have an MR? How did you patients react to the introduction? (positive or negative)

What could be the motivations (for doctors and/or patients) not to participate or leave? Is there too much administrative work involved? Could this be a negative aspect of the programme? Can you provide examples?

What have been the positive and negative aspects (problems) of the programme according to your daily experience? Examples?

Do you think a stronger role for primary care doctors in referring patients to specialist care (i.e. gate-keeping) is required?

Can you describe briefly how the MR scheme works in practice according to your experience? (Communication, formation process…).

Does the doctor or patient first suggest participating in the MR programme? (In general, do you propose participation in the programme to your patients or do your patients ask you about participating in the programme).

How do you and patients obtain information about the programme or know that it exists and how it works?

What is the relation between the MR and other actors such as the Ministry or AMMD? Is there an active promotion of the programme?

How have you implemented the MR scheme? How much time, investment and administrative work does it entail?

In your view as a GP, what is the role of primary care in the health system in Luxembourg compared to other countries? How was the situation of primary care before the MR reform?

What have been the positive and negative aspects (strengths and problems) of the programme according to your daily experience? Examples?

In general, there is low participation of patients in the programme – what are the reasons for this in your opinion?

Are there differences in participation by GPs in the programme across the country (if yes, can probe if this could be linked to patient characteristics e.g. age, health status or socio-economic)?

1. **Link to diabetes (All interviewees)**

We found that the programme was associated with small increases in some care quality indicators for type 2 diabetes. Do you think participating in the MR programme encouraged GPs to change their clinical practice, for example to follow clinical guidelines for diabetes?

For doctors participating in the programme: do you think the care you provided to patients with an MR differed to that provided to patients without an MR?

What are the positive aspects for a patient if treated in this case by MR?

Final question:

Any other information, observation, or theme that you find interesting or important and which could make us better understand the topic…

**S4 Table. Attrition due to deaths, number, sample size and percentage by year**

| **Year** | **Sample size** | **Number of deaths** | **Percentage** |
| --- | --- | --- | --- |
| 2010 | 16,775 | 197 | 1.17 |
| 2011 | 16,578 | 332 | 2.00 |
| 2012 | 16,246 | 418 | 2.57 |
| 2013 | 15,828 | 376 | 2.38 |
| 2014 | 15,452 | 418 | 2.71 |
| 2015 | 15,034 | 417 | 2.77 |
| 2016 | 14,617 | 452 | 3.09 |
| 2017 | 14,165 | 452 | 3.19 |
| 2018 | 13,713 | 450 | 3.28 |

**S5 Table. Additional outcomes difference-in-differences estimates, p-values and 95% confidence intervals**

|  | **Short-term (up to 2015)** | | | | **Medium-term (up to 2018)** | | | |
| --- | --- | --- | --- | --- | --- | --- | --- | --- |
| **Variable** | **Coefficient** | **P-value** | **95% Confidence Interval** | | **Coefficient** | **P-value** | **95% Confidence Interval** | |
| *Utilisation (number per patient per year)* | | | | | | | | |
| Urgent or out-of hours consultations | -0.01 | 0.889 | -0.09 | 0.07 | 0.01 | 0.816 | -0.07 | 0.08 |
| Urgent or out-of hours visits | 0.01 | 0.810 | -0.04 | 0.05 | 0.01 | 0.728 | -0.03 | 0.04 |
| Inpatient hospitalisations | -0.05 | 0.071 | -0.10 | 0.00 | -0.02 | 0.384 | -0.07 | 0.03 |
| Day patient admission | 0.01 | 0.823 | -0.04 | 0.05 | 0.01 | 0.714 | -0.03 | 0.04 |
| *Costs (per patient per year, Euro inflated to 2018 prices)* | | | | | | | | |
| GP costs | 150.81**^** | 0.000 | 138.62 | 162.99 | 149.43**^** | 0.000 | 137.21 | 161.65 |
| Specialist costs | -85.55 | 0.127 | -195.32 | 24.23 | -51.63 | 0.330 | -155.58 | 52.32 |
| *Continuity of care* | | | | | | | | |
| Number of different GPs reimbursed for a patient | 0.04 | 0.126 | -0.01 | 0.08 | 0.01 | 0.594 | -0.03 | 0.05 |

**^**The difference-in-differences assumption of pre-intervention parallel trends does not hold

**S6 Table. Difference-in-differences estimates, p-values and 95% confidence intervals, including patients with deliveries of insulin in addition to oral medication**

|  | **Short-term (up to 2015)** | | | | **Medium-term (up to 2018)** | | | |
| --- | --- | --- | --- | --- | --- | --- | --- | --- |
| **Variable** | **Coefficient** | **P-value** | **95% Confidence Interval** | | **Coefficient** | **P-value** | **95% Confidence Interval** | |
| *Utilisation (number per patient per year)* | | | | | | | | |
| GP consultations | 0.52^ | 0.000 | 0.31 | 0.74 | 0.31^ | 0.004 | 0.10 | 0.52 |
| GP home visits | 0.07 | 0.110 | -0.02 | 0.15 | 0.07 | 0.082 | -0.01 | 0.16 |
| Specialist consultations | -0.15 | 0.201 | -0.37 | 0.08 | -0.10 | 0.364 | -0.31 | 0.12 |
| Cardiologist consultations | -0.03 | 0.242 | -0.08 | 0.02 | -0.02 | 0.371 | -0.07 | 0.03 |
| *Care process indicators, number per patient per year* | | | | | | | | |
| HbA1c tests | 0.15^ | 0.001 | 0.07 | 0.24 | 0.14^ | 0.001 | 0.06 | 0.22 |
| Complete cholesterol test | 0.04^ | 0.255 | -0.03 | 0.11 | 0.06^ | 0.057 | -0.00 | 0.12 |
| Kidney function (blood) test | 0.20^ | 0.000 | 0.11 | 0.29 | 0.17^ | 0.000 | 0.09 | 0.26 |
| Kidney function (urine) test | 0.07 | 0.002 | 0.03 | 0.11 | 0.07 | 0.001 | 0.03 | 0.11 |
| Eye exam by an ophthalmologist | 0.04 | 0.354 | -0.04 | 0.12 | 0.04 | 0.330 | -0.04 | 0.12 |
| Dental consultation | 0.06 | 0.039 | 0.00 | 0.11 | 0.04 | 0.108 | -0.01 | 0.09 |
| *Prescribed medicines, number per patient per year* | | | | | | | | |
| Total repeat (>3 deliveries) prescribed medicines | 0.19 | 0.014 | 0.04 | 0.35 | 0.20 | 0.009 | 0.05 | 0.36 |
| Repeat ATC B – Blood and blood forming organs | 0.02 | 0.149 | -0.01 | 0.05 | 0.00 | 0.795 | -0.03 | 0.03 |
| Repeat ATC C – Cardiovascular system | 0.04^ | 0.255 | -0.03 | 0.01 | 0.04^ | 0.167 | -0.02 | 0.11 |
| Repeat ATC D - Dermatologicals | -0.01 | 0.674 | -0.03 | 0.02 | -0.01 | 0.241 | -0.03 | 0.01 |
| Repeat ATC N - Nervous system | 0.05^ | 0.081 | -0.01 | 0.10 | 0.05^ | 0.076 | -0.01 | 0.10 |
| Repeat ACT S – Sensory organs | 0.01 | 0.490 | -0.01 | 0.03 | 0.01^ | 0.204 | -0.01 | 0.03 |

**^**The difference-in-differences assumption of pre-intervention parallel trends does not hold

**S7 Table. Difference-in-differences estimates, p-values and 95% confidence intervals, using patients not yet treated as the control group**

|  | **Short-term (up to 2015)** | | | | **Medium-term (up to 2018)** | | | |
| --- | --- | --- | --- | --- | --- | --- | --- | --- |
| **Variable** | **Coefficient** | **P-value** | **95% Confidence Interval** | | **Coefficient** | **P-value** | **95% Confidence Interval** | |
| *Utilisation (number per patient per year)* | | | | | | | | |
| GP consultations | 0.34^ | 0.004 | 0.11 | 0.57 | 0.20^ | 0.076 | -0.02 | 0.43 |
| GP home visits | 0.02 | 0.602 | -0.05 | 0.08 | 0.06^ | 0.136 | -0.02 | 0.13 |
| Specialist consultations | -0.28 | 0.021 | -0.51 | -0.04 | -0.26 | 0.025 | -0.49 | -0.03 |
| Cardiologist consultations | -0.01 | 0.773 | -0.06 | 0.05 | 0.02 | 0.521 | -0.04 | 0.07 |
| *Care process indicators, number per patient per year* | | | | | | | | |
| HbA1c tests | 0.08^ | 0.072 | -0.01 | 0.17 | 0.10 | 0.022 | 0.01 | 0.18 |
| Complete cholesterol tests | -0.01^ | 0.850 | -0.08 | 0.07 | 0.01 | 0.821 | -0.06 | 0.08 |
| Kidney function (blood) tests | 0.11 | 0.017 | 0.02 | 0.19 | 0.11 | 0.014 | 0.02 | 0.19 |
| Kidney function (urine) tests | 0.05^ | 0.044 | 0.00 | 0.09 | 0.05 | 0.021 | 0.01 | 0.10 |
| Eye exam by an ophthalmologist | -0.02 | 0.719 | -0.10 | 0.07 | -0.02 | 0.725 | -0.10 | 0.07 |
| Dental consultation | 0.03 | 0.358 | -0.03 | 0.09 | 0.01 | 0.640 | -0.04 | 0.07 |
| *Prescribed medicines, number per patient per year* | | | | | | | | |
| Total repeat (>3 deliveries) prescribed medicines | 0.18 | 0.030 | 0.02 | 0.34 | 0.17 | 0.039 | 0.01 | 0.33 |
| Repeat ATC B – Blood and blood forming organs | 0.01 | 0.375 | -0.02 | 0.04 | 0.02 | 0.257 | -0.01 | 0.05 |
| Repeat ATC C – Cardiovascular system | 0.06 | 0.051 | 0.00 | 0.12 | 0.08 | 0.022 | 0.01 | 0.14 |
| Repeat ATC D - Dermatologicals | -0.01 | 0.398 | -0.04 | 0.02 | -0.02 | 0.144 | -0.04 | 0.01 |
| Repeat ATC N - Nervous system | 0.05 | 0.083 | -0.01 | 0.11 | 0.05 | 0.088 | -0.01 | 0.11 |
| Repeat ACT S – Sensory organs | 0.00 | 0.982 | -0.02 | 0.02 | -0.01 | 0.493 | -0.03 | 0.01 |

**^**The difference-in-differences assumption of pre-intervention parallel trends does not hold
